# The effects of chemical and organic fertilizer usage on rhizosphere soil in tea orchards

**DOI:** 10.1371/journal.pone.0217018

**Published:** 2019-05-28

**Authors:** Weiwei Lin, Manhong Lin, Hongyan Zhou, Hongmiao Wu, Zhaowei Li, Wenxiong Lin

**Affiliations:** 1 Fujian Provincial Key Laboratory of Agroecological Processing and Safety Monitoring, College of Life Sciences, Fujian Agriculture and Forestry University, Fuzhou, China; 2 Key Laboratory of Crop Ecology and Molecular Physiology, Fujian Agriculture and Forestry University, Fuzhou, China; 3 Key Laboratory for Genetics, Breeding and Multiple Utilization of Crops, Ministry of Education / College of Crop Science, Fujian Agriculture and Forestry University, Fuzhou, China; 4 Fujian Vocational College of Agriculture, Fuzhou, China; Sichuan Agricultural University, CHINA

## Abstract

Sustainable agriculture is an important global issue. The use of organic fertilizers can enhance crop yield and soil properties while restraining pests and diseases. The objective of this study was to assess the effects of long-term use of chemical and organic fertilizers on tea and rhizosphere soil properties in tea orchards. Inductively coupled plasma mass spectrometry (ICP-MS) and high-throughput sequencing technology analyses were used to investigate heavy metals content and bacterial composition in rhizosphere soils. Our results indicated that organic fertilizer treatment significantly decreased Cu, Pb and Cd contents in rhizosphere soil sample. The results also showed that treatment with organic fertilizer significantly decreased the contents of Cd, Pb and As in tea leaves. Furthermore, organic fertilizer significantly increased the amino acids content of tea and the pH of the soil. The use of organic fertilizer significantly increased in the relative abundance of *Burkholderiales*, *Myxococcales*, *Streptomycetales*, *Nitrospirales*, *Ktedonobacterales*, *Acidobacteriales*, *Gemmatimonadales*, and *Solibacterales*, and decreased the abundance of *Pseudonocardiales*, *Frankiales*, *Rhizobiales*, and *Xanthomonadales*. In conclusion, organic fertilizer can help to shape the microbial composition and recruit beneficial bacteria into the rhizosphere of tea, leading to improved tea quality and reduced heavy metals content in rhizosphere soil and tea leaves.

## Introduction

Camellia sinensis, commonly known as tea plant, is a mountainous crop and an important agricultural product for many farmers in China. Due to its richness in beneficial antioxidants, vitamins, and amino acids, the popularity of tea has been steadily growing. And the amino acids, tea polyphenols, and caffeine are key elements in determining both taste and quality of tea. In 2016, China produced 2.41 million tons of tea with a monetary value of 170.2 billion yuan. However, soil degradation and substantial quality and yield decrease have been observed in the long-term monoculture of tea bushes, and have become key problems in the sustainable development of tea orchards [1]. With growing demand and limited land availability, farmers have been increasingly using nitrogen fertilizers in order to increase crop yield. However, nitrogen fertilizers can have undesirable effects, including a decline in tea quality, soil acidification, heavy metals pollution, soil compaction, and changes in soil microbiome [2–3]. We have previously shown that long-term tea cultivation with nitrogen fertilizers altered the bacterial composition of soil and significantly decreased soil pH and microbial metabolic activity, resulting in a reduction of beneficial bacteria [1, 4–5]. Therefore, it is essential to understand the impact of nitrogen fertilizers beyond their effects on crop yield in order to achieve a balance between benefits and harms in modern agricultural practices.

The sustainability of agricultural systems is an important global issue. This has resulted in the potential benefits of organic fertilizers application have being highlighted. Organic fertilizers are derived from natural sources (e.g., livestock and poultry excreta, plant residues, biogas residue, and agricultural by-products), and their usage can have a positive impact on pollution. The potential benefits of organic fertilizers have been documented in a number of studies in which investigators observed a raise in soil microbial activities, which in turn improved crop growth and restrained pests and diseases [6–7]. Soil contains a large number of microbial species as well as other organisms that together form a highly complex ecosystem. Microorganisms are essential for nutrient recycling, healthy plant development, and decomposition of organic matter [8]. However, environmental conditions and cultivation practices are likely to influence the microbiome, resulting in alterations in soil characteristics or ecosystem [9]. Researchers have found that tea cultivated with bio-organic fertilizers has superior color and taste compared to tea treated with chemical fertilizers [10–11]. Studies have also suggested that the use of organic fertilizers resulted in higher seedling biomass and significantly improved the soil fungal to bacterial ratio as well as soil enzyme activity [12–13]. In addition, while long-term application of chemical fertilizers could lead to serious soil acidification, nutritional imbalance, and deterioration of the rhizosphere micro-ecological environment, further increased the activity of heavy metal ions in soil. The use of organic fertilizer could alleviate soil acidification, resulting in increased plant yields [14]. However, little information is available concerning the contents changes of microbial community and heavy metal ions using long-term chemical fertilizers and organic fertilizer.

Recent studies have raised concerns regarding the long-term effects of fertilization practices on biological properties of soils, but most studies focused mainly on tea plant yield and changes in soil nutrients [15–17]. Moreover, the effects of long-term use of organic fertilizer on rhizosphere bacterial composition and heavy metals in tea gardens have not been carefully investigated. In this study, we have used ICP-MS and high-throughput sequencing technology to determine the effects of organic and chemical fertilizers on bacterial taxa and heavy metals content in the rhizosphere of tea orchards. Herein we evaluated potential mechanisms of organic fertilizers application as a means to improve the quality of tea. These results could provide practical guidance to the design of sustainable tea garden ecology.

## Materials and methods

### Field experiment and soil sampling

The experimental station is located in the Zudun township of Nanping in the Fujian Province of China (27°24ʹN, 118°33ʹE). This is one of the main tea producing region in Fujian and is under sub-tropical monsoon climate with an average annual temperature of 18.1°C and an average annual rainfall of 1557–1743 mm. Zudun township of Fujian Province is the most important producing area of white tea in China. The two different planting and management patterns were the long-term organic fertilizer (mainly contain rape cake and sheep dung) ecological tea garden and the conventional nitrogen fertilizer management tea garden. These tea orchards had similar environmental characteristics such as altitute, slope position and slope aspect, and similar agronomic management. The tea orchards were typical red acidity soils in southern China. These experimental fields were established in 1990 and have since been used for tea planting. The tea orchards have cultivated the Fuding white tea and the tea stand ages were more than 30 years old, were selected in this study, and each had three landscape-level replications. The organic tea orchards have been certified under the US National Organic program 7 CFR Part 205 by the certification of environmental standards Gmbh (CERES) in 2015.

Soil samples were collected from the rhizosphere of fields treated with organic fertilizer (OrgS) or chemical fertilizer (NorS) fon June 4, 2018. At the same time, non-rhizosphere soils were also collected from the organic (CKOrgS) and chemical (CKNorS) fertilizer treated tea orchards. The rhizosphere and non-rhizosphere soils of tea trees were taken from each experimental plot by a 5-point sampling method. For each sample, three replicates were performed. Tea leaves from the OrgS and NorS treatment groups were sampled in the fields. After sieving (2 mm mesh) to remove stones and plant residues, soil samples were stored at −80°C.

### Analysis of tea biochemical properties

Tea leaves were roasted, dried and ground into a fine powder testing. The amino acid content was measured using the ninhydrin colorimetric methods. Tea polyphenols and caffeine contents were determined using a Waters HPLC system (C18 column: Inertsil ODS-SP, 4.6 × 250 mm, 5 μm). The chromatographic conditions were as follows for tea polyphenols: mobile phase A: mixture solution (water: acetic acid: acetonitrile = 90:0.1:10, v/v/v); mobile phase B: acetonitrile; elution gradient: mobile phase B 0% (0 min)→0% (10 min)→10% (20 min)→0% (25 min)→0% (30 min); oven temperature: 40°C; detection wavelength: 280 nm; velocity: 1 mL/min. For caffeine: mobile phase A: water; mobile phase B: methanol; elution gradient: mobile phase B 65% (0 min)→65% (35 min); detection wavelength: 275 nm; velocity: 1 mL/min.

### Analysis of soil chemical properties

Soil chemical properties analysis included pH, total nitrogen (TN), total phosphorus (TP), total potassium (TK), available nitrogen (AN), available phosphorus (AP), and available potassium (AK). Soil pH was determined using a glass electrode pH meter (1:2.5 soil to water suspensions). TN, TP, and TK were determined using Kjeldahl digestion, sodium carbonate fusion, and NaOH melts flamer methods, respectively [18]. AN was determined using the alkaline hydrolyzable method. AP was extracted with hydrochloric acid and ammonium fluoride, and contents were measured using the molybdenum blue method. AK was extracted with ammonium acetate, and measured by flame photometry [19].

### Analysis of heavy soil metal

The microwave digestion system (Milestone ETHOS UP, Italy) was used to extract cuprum (Cu), plumbum (Pb), cadmium (Cd) and arsenic (As) from soil samples. The contents of these metals were determined by Inductively coupled plasma mass spectrometry (ICP-MS, PerkinElmer NexION 300X, U.S.A), using parameters listed in Table 1.

**Table 1:** The parameters for the ICP-MS.

As	Cd	Pb	Cu	Parameters
Radio-frequency power		1300 w		
Plasma velocity		13.00 L/min		
Auxiliary airflow velocity		1.40 L/min		
Flow rate of the carrier gas		0.95 L/min		
Mass-to-charge ratio (m/z)	63	208	111	75
Atomization device		MCN		
Atomizer chamber		Peltier-cooler (2°C)		

### Genomic DNA purification and PCR amplification

Total soil DNA was extracted using the BioFast soil Genomic DNA Extraction kit (BioFlux, Hangzhou, China), following the manufacturer’s instructions. For each soil sample, three independent DNA extractions were performed. DNA was diluted to a concentration of 1 ng/μL in sterile water. The variable regions 3 to 4 (V3–V4) were amplified with the specific primers 338F/806R (338F, 5’-ACTCCTACGGGAGGCAGCA-3’; 806R, 5’-GGACTACHVGGGTWTCTA AT-3’). The PCR reactions were conducted in a 50 μL mixture system, using TransStart Fastpfu DNA Polymerase (TransGen Biotechnology, Beijing, U.S.A). The PCR condition was initiated denaturation with 5 min at 95°C, followed by 35 cycles of 40 s at 95°C, 40 s at 58°C, 60 s at 72°C and final elongation with 5 min at 72°C. PCR products were purified using the Qiagen Gel Extraction Kit (Qiagen, Germany), and subjected to sequencing on the Illumina HiSeq 2500 platform (Allwegene Technologies Co., Ltd., Beijing, China).

### Statistical analysis

The FLASH method [20] was used to merge paired-end reads. Following quality filtering and chimera removal [21], the effective tags were used to perform OTU clustering and species annotation. Species annotation was performed using the Silva database (http://www.arb-silva.de) [22]. For each remaining sequences, the RDP classifier (Version 2.2) algorithm [23] was used to annotate taxonomic information via the GreenGen database [24]. Mothur version 1.31.2 [25] was used to analyze the alpha diversities. Beta diversities were then calculated to analyze the complexity of species diversity. The Statistical Package for the GraphPad Prism version 5.1 and the Data Processing System (DPS) version 7.05 were used for statistical analysis. Differences among the treatments were calculated and statistically analyzed using the analysis of variance (ANOVA) and the LSD multiple range tests (p < 0.05).

## Results

### Tea and soil chemical characteristics

**Fig 1:**
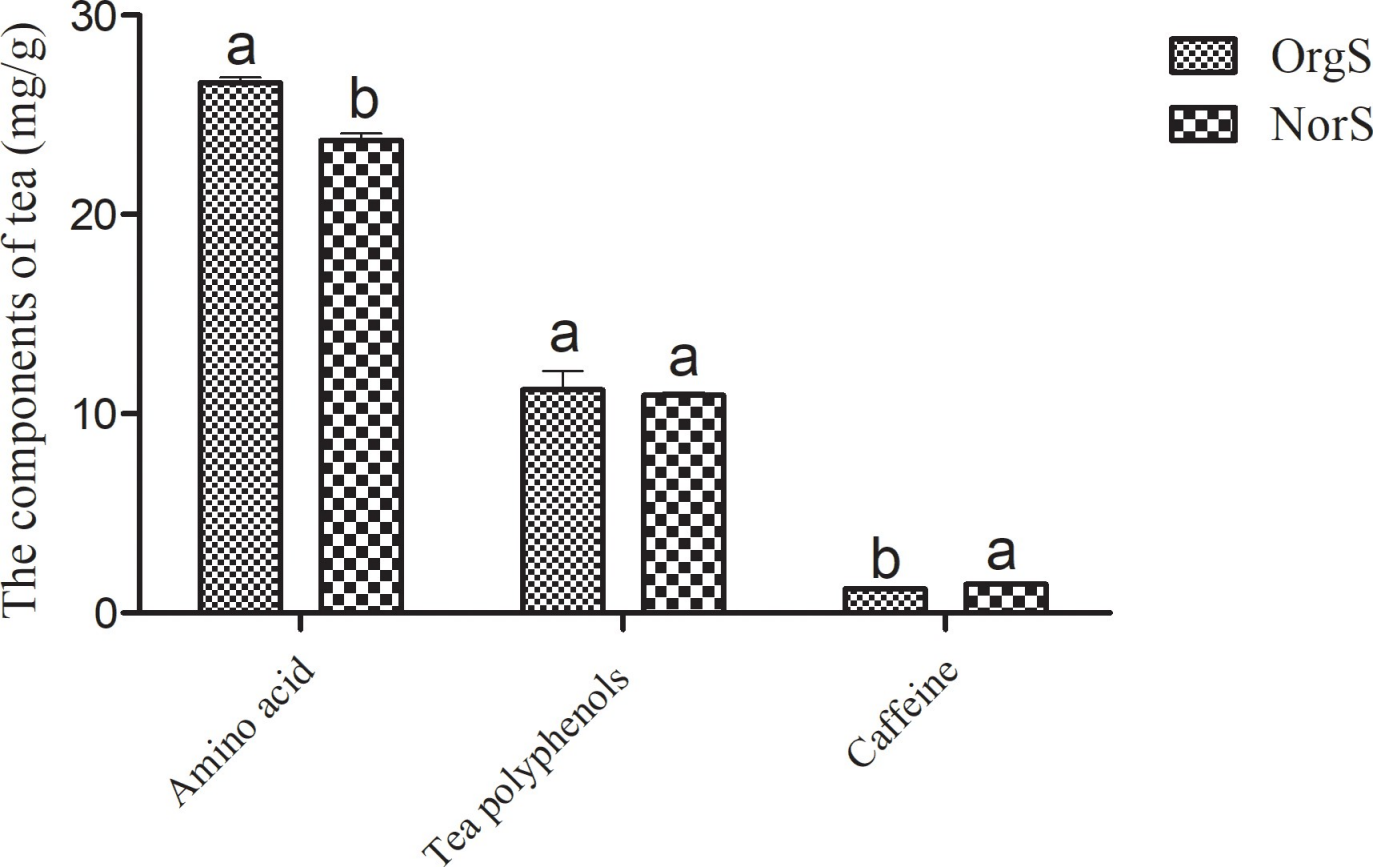
Amino acids, polyphenols, and caffeine contents of tea under treatment with organic (OrgS) or chemical (NorS) fertilizer. Columns with different letters are statistically different (LSD test, *P* < 0.05).

In this study, we found that the use of organic fertilizer significantly increased the amino acids content of tea (Fig 1). The contents of polyphenol did not reach statistical significance in tea samples from fields treated with organic fertilizer compared to those treated with chemical fertilizer. In contrast, tea under long-term treatment with chemical fertilizer showed significantly higher contents of caffeine compared to tea with organic fertilizer.

Table 2 summarized the chemical properties of soil from tea orchards treated with either organic or chemical fertilizers. Contents of total nitrogen, total potassium, available nitrogen, available phosphorus, and available potassium were similar between the two treatment groups (P > 0.05). However, soil pH level was significantly higher in the organic fertilizer treatment group compared to the chemical fertilizer treatment group (Table 2).

**Table 2:** Chemical properties of soils from tea orchards with different treatments.

Soil chemical properties	CKOrgS	CKNorS	OrgS	NorS
Total nitrogen (TN) (g/kg)	1.06b	1.38ab	1.59ab	1.82a
Total phosphorus (TP) (g/kg)	0.27b	0.22b	0.36b	1.60a
Total potassium (TK) (g/kg)	6.16a	4.73a	6.42a	5.47a
Available nitrogen (AN) (g/kg)	0.08b	0.13ab	0.16ab	0.18a
Available phosphorus (AP) (g/kg)	0.01b	0.01b	0.03ab	0.08a
Available potassium (AK) (g/kg)	0.11a	0.10a	0.13a	0.13a
pH	4.24a	4.10ab	4.19a	4.00b

OrgS and NorS refer to rhizosphere soils of organic fertilizer and chemical fertilizer treatments, respectively. CKOrgS and CKNorS refer to non-rhizosphere soils. Columns with different letters are statistically different (LSD test, P < 0.05).

**Fig 2:**
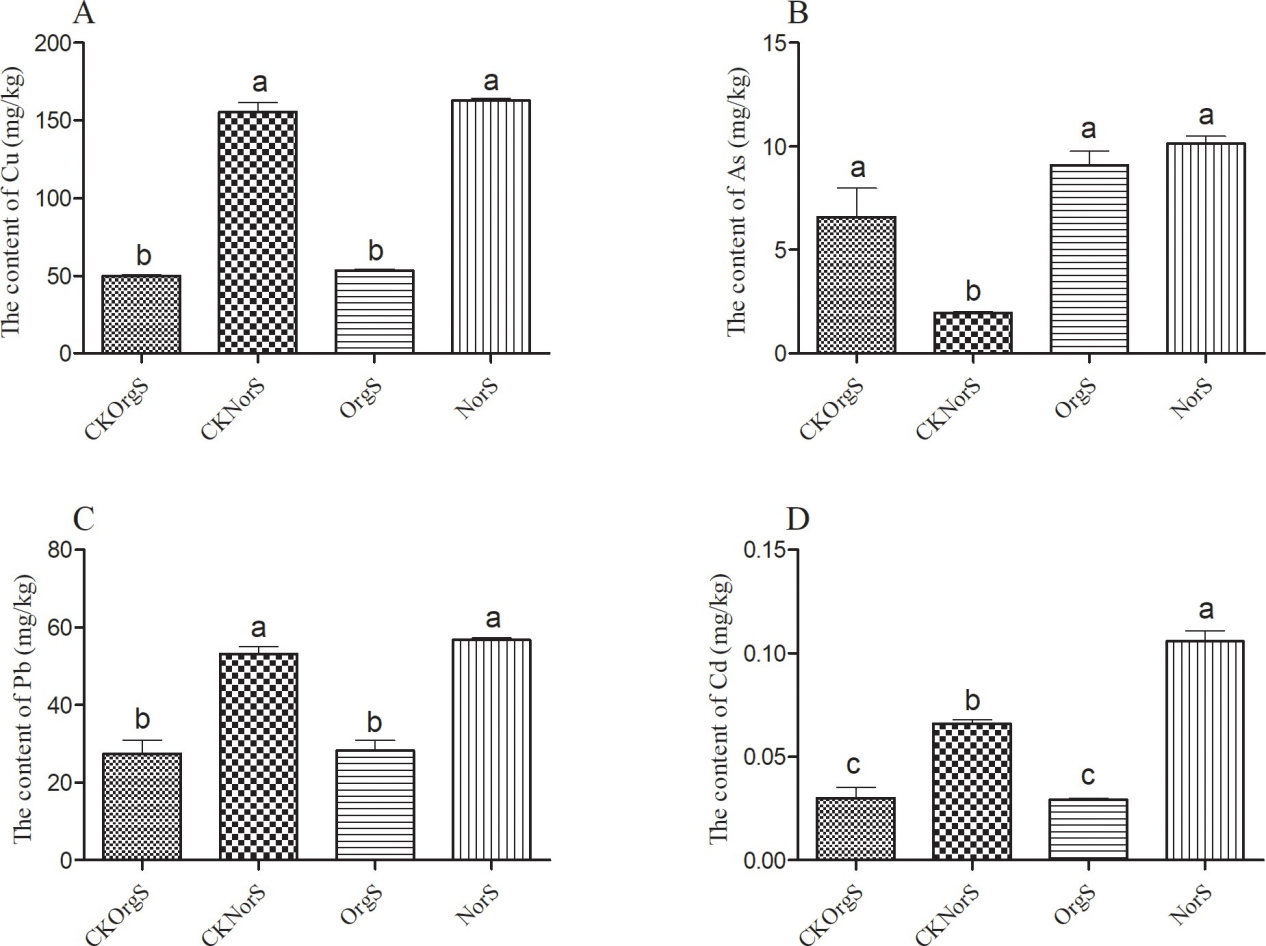
Heavy metals content in non-rhizosphere and rhizosphere soil samples from tea orchards under organic or chemical fertilizer treatment. Columns with different letters are statistically different (LSD test, *P* < 0.05).

**Fig 3:**
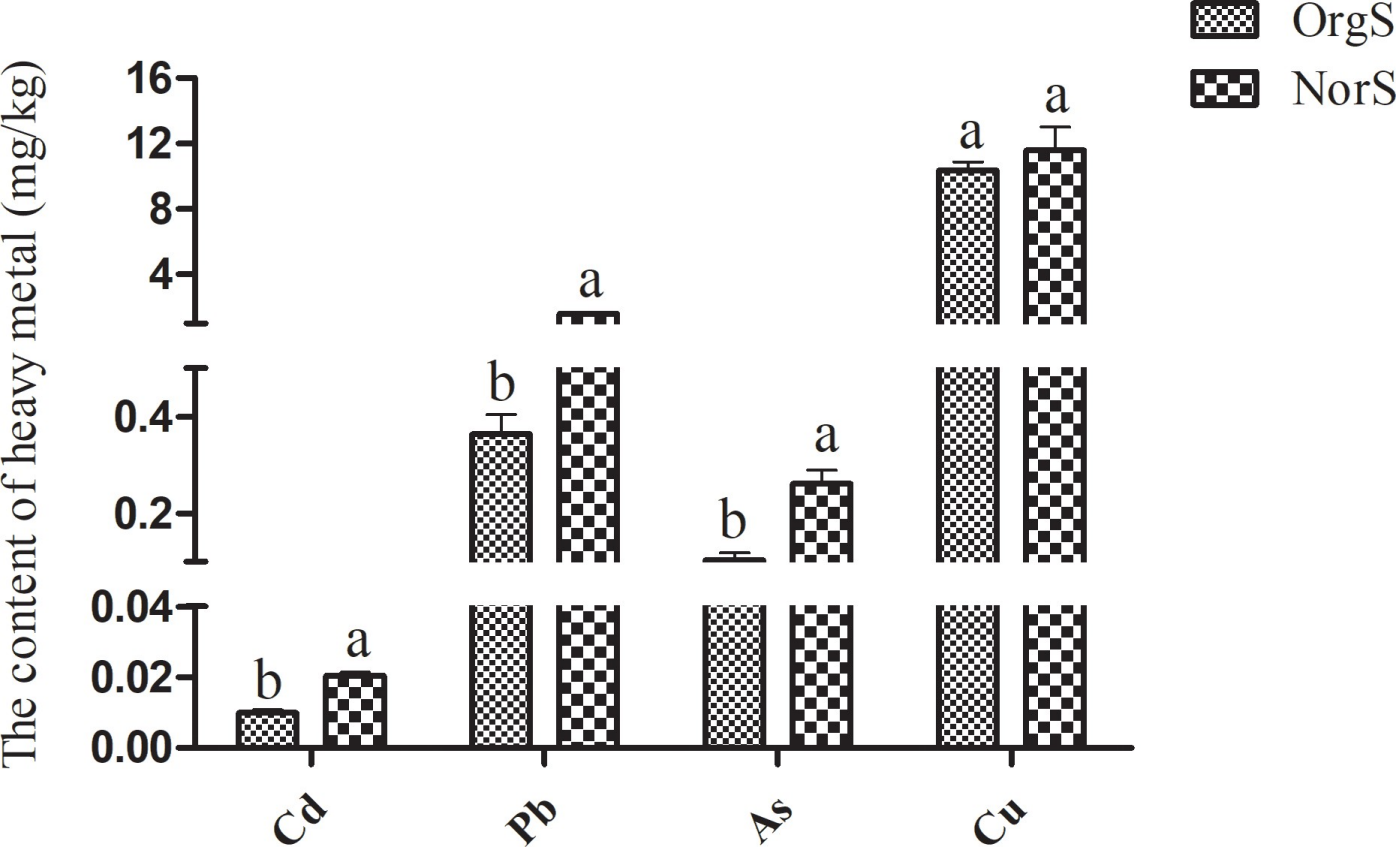
Heavy metals content in tea leaf samples from tea orchards under organic or chemical fertilizer treatment. Columns with different letters are statistically different (LSD test, *P* < 0.05).

Tea orchards with long-term organic or chemical fertilizer treatment showed significant differences in soil chemical properties (Fig 2). Treatment with organic fertilizer resulted in significantly lower contents (P < 0.05) of cuprum (Cu), plumbum (Pb) and cadmium (Cd) in rhizosphere soils compared to the chemical fertilizer treatment group. A small decrease in arsenic (As) level was also detected in the organic fertilizer treatment group, but the difference was not statistically significant. Similar trends were observed in non-rhizosphere soil samples. Our results also showed that treatment with organic fertilizer significantly decreased contents of Cd, Pb and As in tea leaves (Fig 3).

### Alpha diversity indices of microbial community

**Fig 4:**
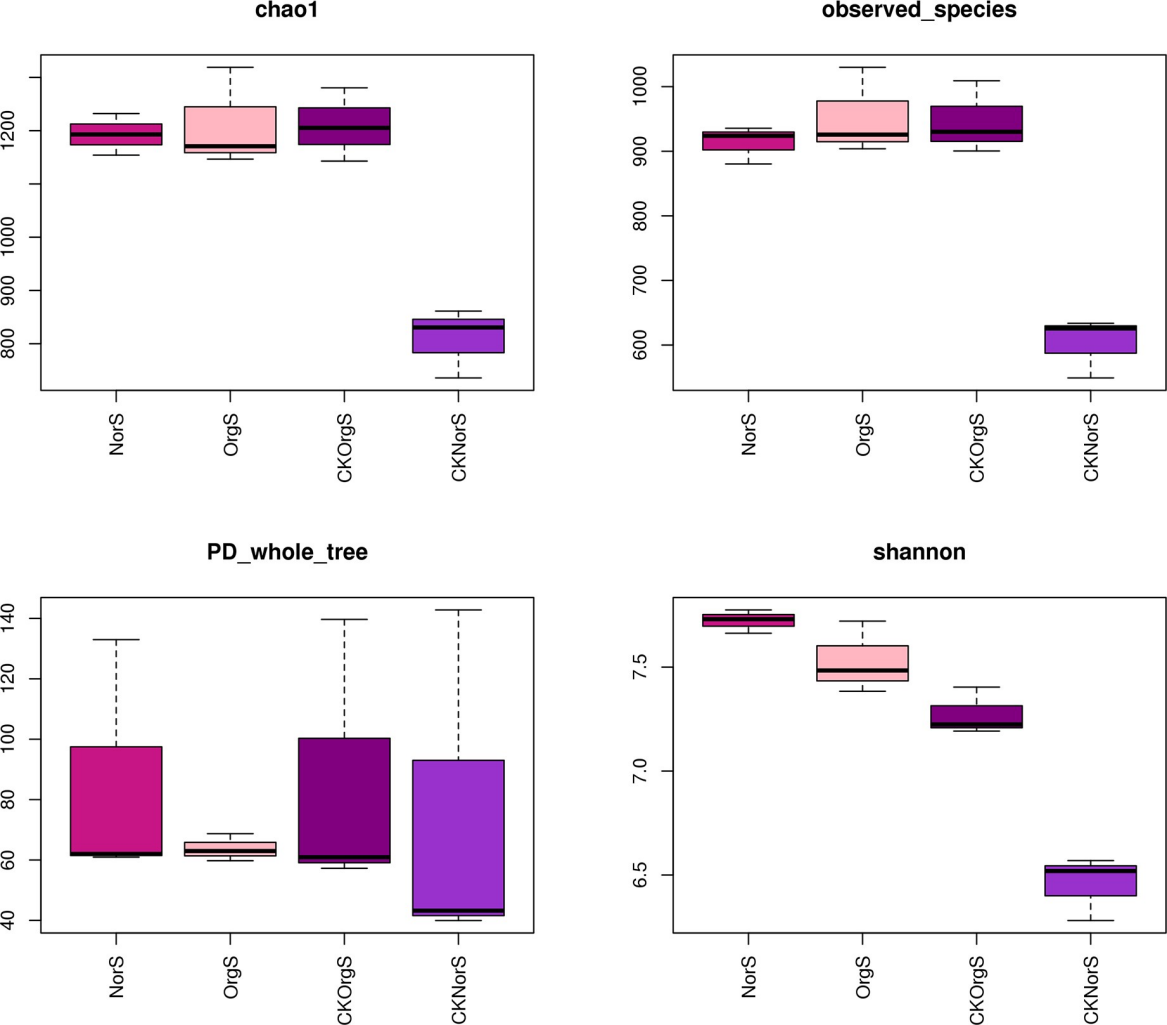
Observed richness, OTUs and diversity of soil samples from organic and chemical fertilizer treatment groups.

A total of 544,096 effective clean tags with bacterial species annotation were obtained from 12 soil samples. Alpha diversity was calculated to determine the complexity of species diversity. We observed a significantly higher bacterial composition and Chao1 indices with samples from the organic fertilizer treatment group compared to samples from the chemical fertilizer treatment group. Long-term organic fertilizer treatment also had a positive effect on non-rhizosphere soil. Our results showed that chemical fertilizer significantly increased Shannon’s diversity indices in rhizosphere soil in comparison to all treatments (Fig 4).

### Beta diversity indices of microbial composition

**Fig 5:**
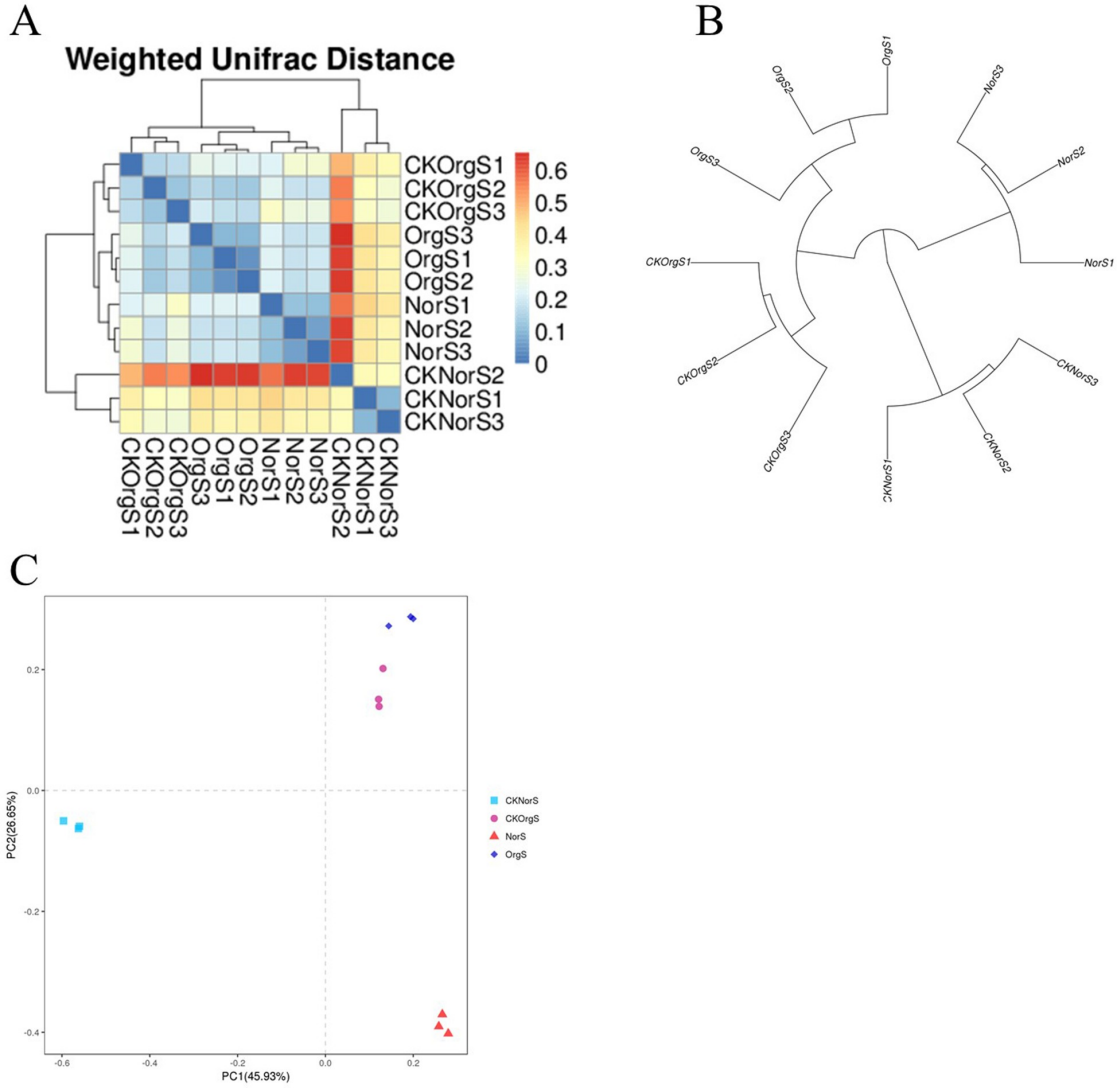
Beta diversity analysis of microbial composition. (A) Weighted unifrac heatmap; (B) Hierarchical clustering analysis; (C) Principal Component Analysis.

We used weighted unifrac heatmap, hierarchical clustering, and principal component analysis to identify differences in bacterial composition structure between the treatment groups (Fig 5). In comparison to CKNorS, higher distances were observed among the OrgS, CKOrgS, and NorS samples. The PC1 and PC2 components of PCoA accounted for 45.93% and 26.65% of the total bacterial composition variations, respectively. We found that the bacterial composition of OrgS and CKOrgS soil samples belonged to the same group based on the principal component analysis. In contrast, the bacterial composition of NorS and CKNorS samples fell into two separate groups that were distinct from OrgS and CKOrgS samples.

### Shifts in soil bacterial composition structure

**Fig 6:**
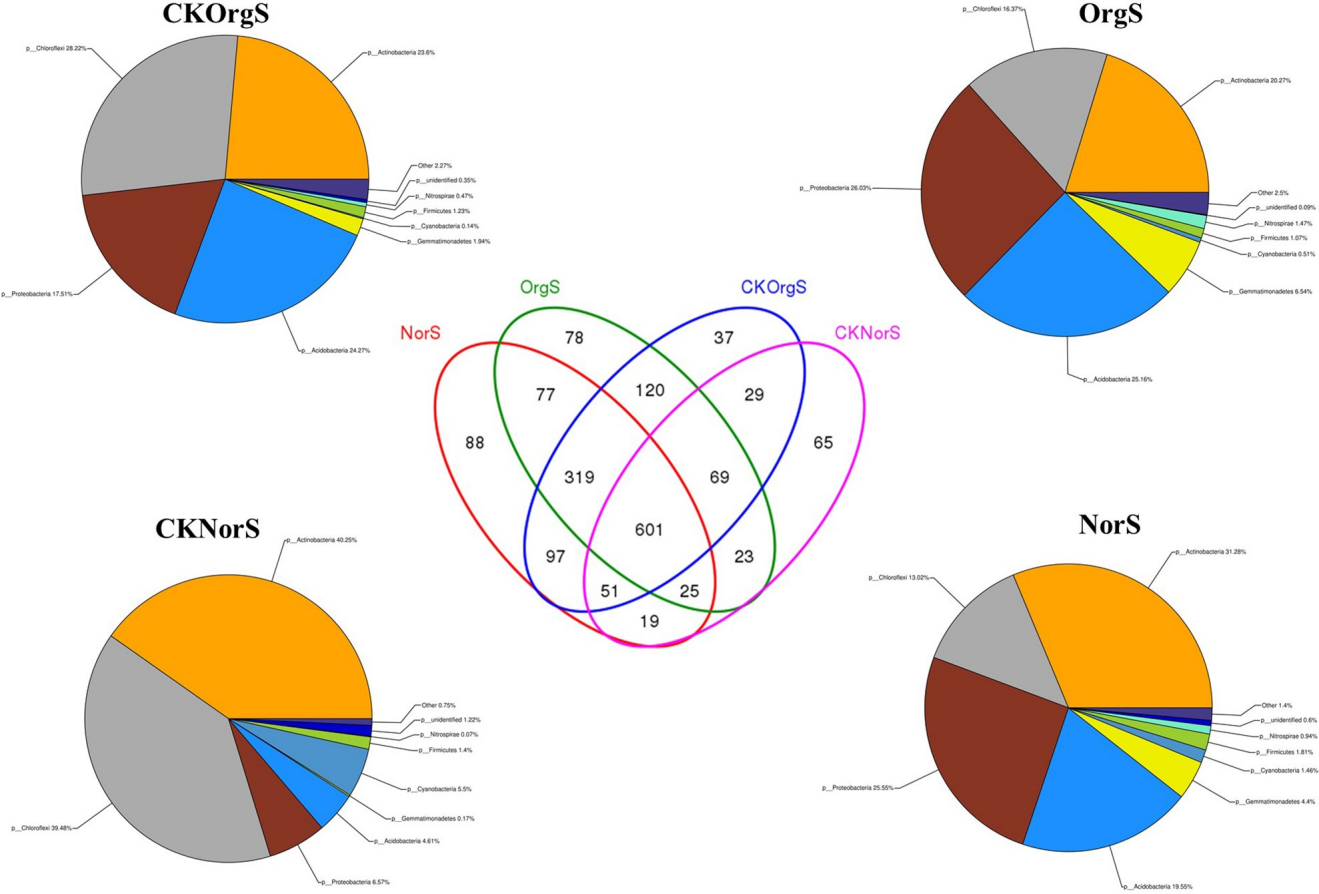
Venn diagram and relative percentages of bacterial phyla in the four different soil samples.

In this study, the classified sequences were affiliated with 24 bacterial phyla among the treatment groups. The majority of the phyla were assigned to *Actinobacteria*, *Chloroflexi*, *Proteobacteria*, *Acidobacteria*, *Gemmatimonadetes*, and *Cyanobacteria* (S1 Fig). Meanwhile, clear trends in variation at the phylum level were observed between the organic fertilizer and chemical fertilizer treatment groups. The number of OTUs exclusively found in OrgS and NorS samples were 78 (4.59%) and 88 (5.18%), respectively. The shared number of exclusive OTUs between OrgS and NorS were 1022 (60.19%). The shared number between OrgS and CKOrgS were 1109 (65.31%), and they dropped to 696 (41.10%) between NorS and CKNorS (Fig 6).

**Fig 7:**
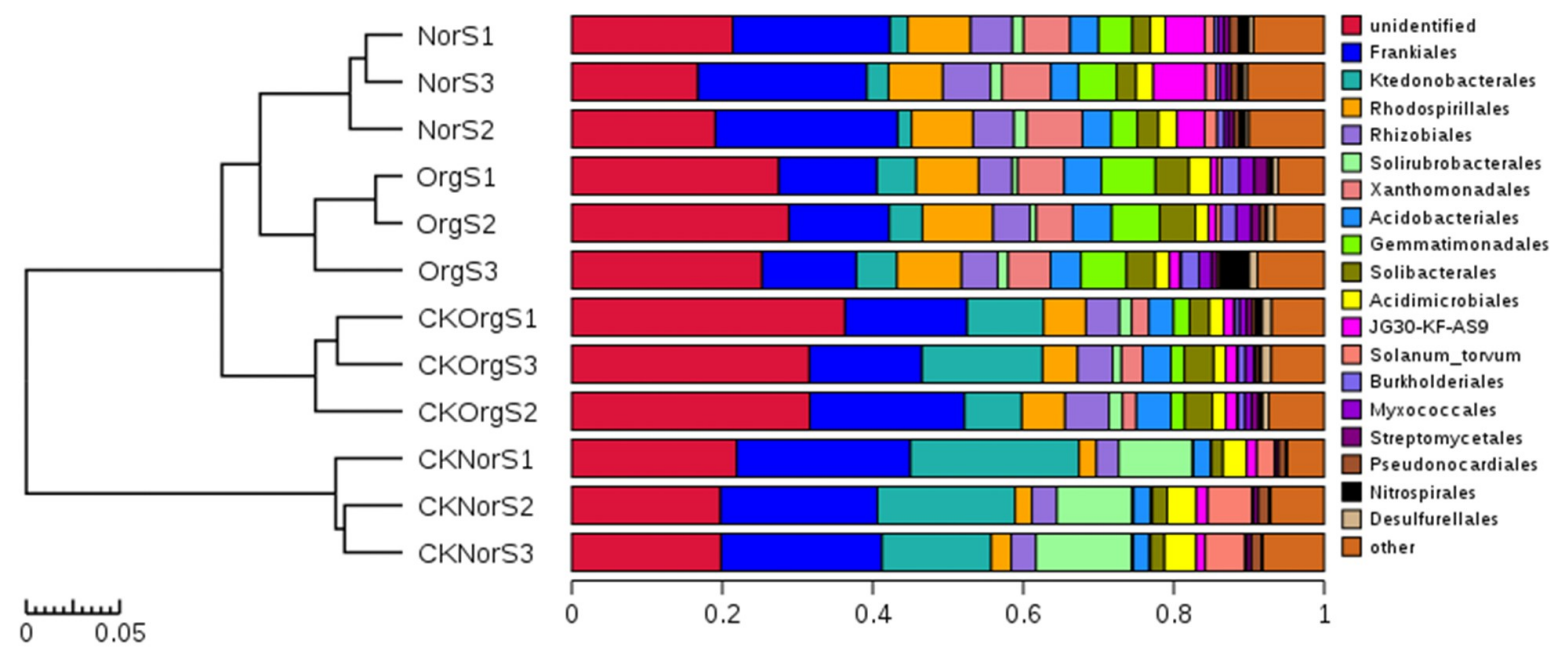
Relative abundance of the top 20 bacterial orders in the four different soil samples.

**Fig 8:**
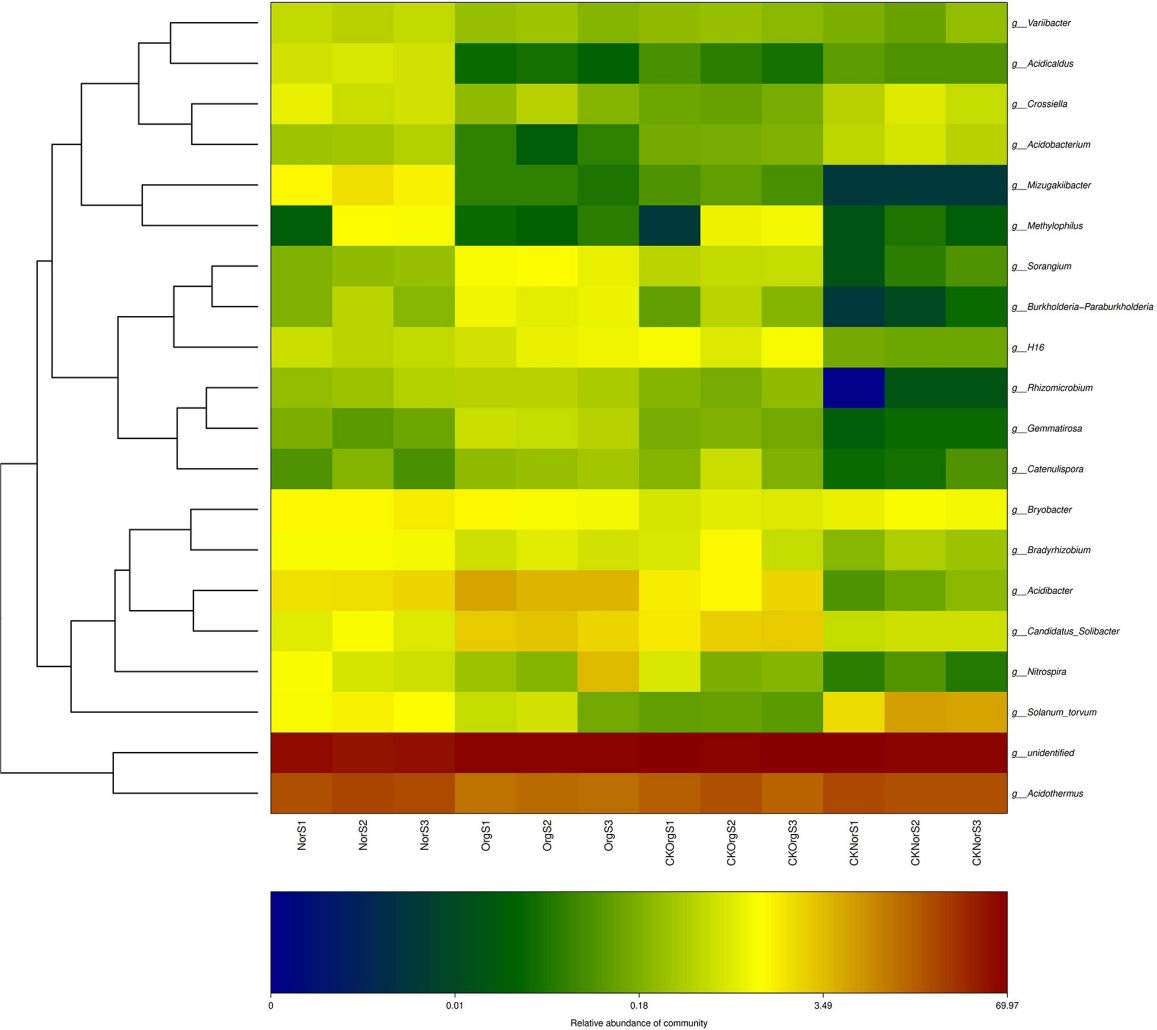
Heat map analysis of the top 20 bacterial orders in the four different soil samples.

The relative abundance of these bacterial orders varied among the different soil samples. A comparison between OrgS and NorS showed that organic fertilizer treatment resulted in a significant increase in the relative abundance of *Burkholderiales*, *Myxococcales*, *Streptomycetales*, *Nitrospirales*, *Ktedonobacterales*, *Acidobacteriales*, *Gemmatimonadales*, and *Solibacterales*, and a decrease in *Pseudonocardiales*, *Frankiales*, *Rhizobiales*, and *Xanthomonadales* (S1 Table and Fig 7). Heat map analysis of the top 20 most abundant genera within the hierarchical cluster showed clear variations in bacterial composition structure across the four groups of soil samples, and these differences were statistically significant. Treatment with chemical fertilizer resulted in increased abundance of *Acidothermus*, *Acidicaldus*, and *Acidobacterium*, and decreased abundance of the potentially beneficial *Nitrospira* and *Burkholderia* in comparison with the organic fertilizer treatment group. No significant differences were detected in comparisons between OrgS and CKOrgS groups, as well as between NorS and CKNorS groups (Fig 8).

### Effects of soil chemical properties on dominant genera

**Fig 9:**
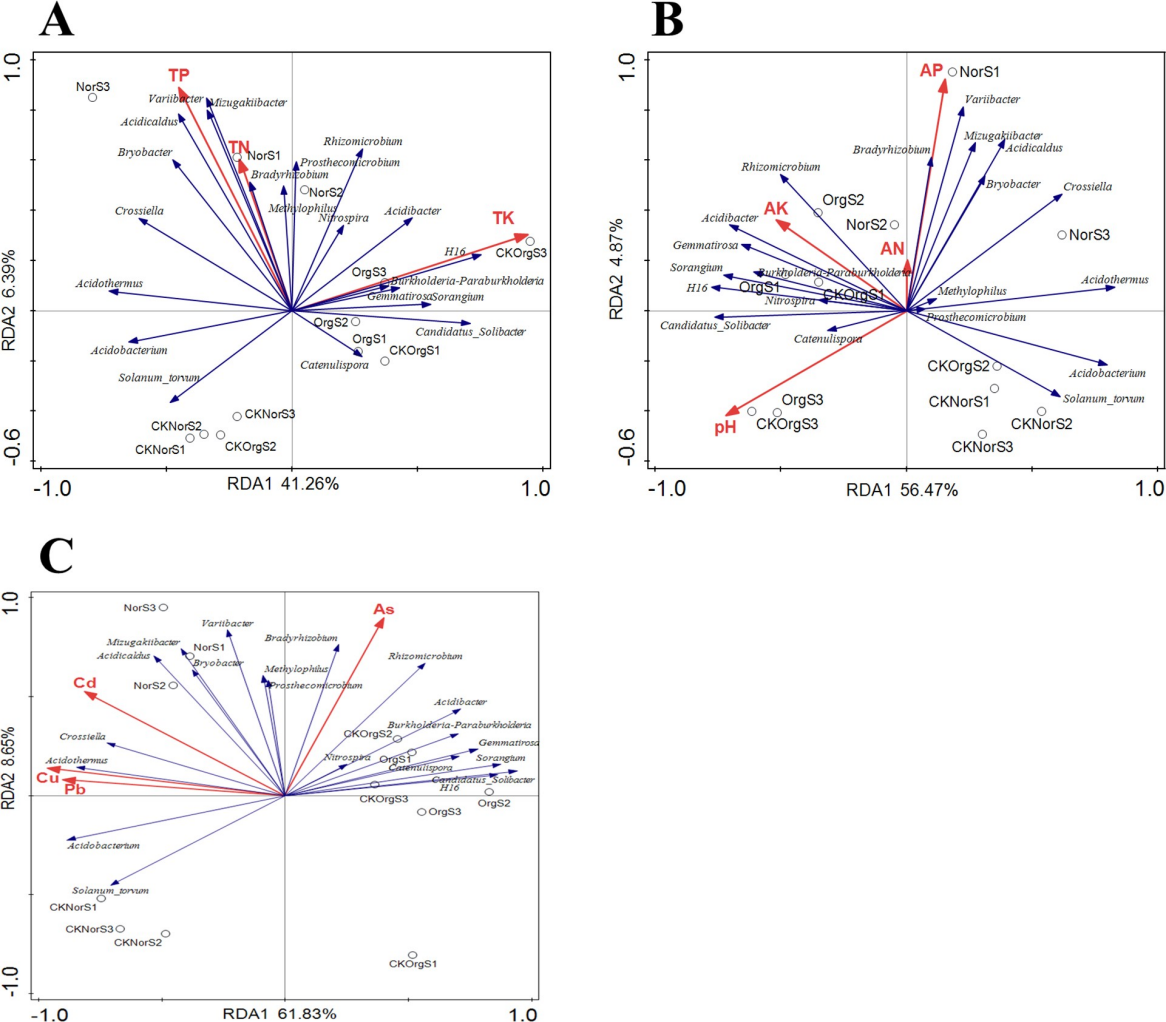
Redundancy analysis (RDA) of the correlation between the most abundant genera of bacteria and soil physiochemical properties. TP: total phosphorus, TN: total nitrogen, TK: total potassium, AN: available nitrogen, AK: available potassium, AP: available phosphorus.

Redundancy analysis (RDA) was performed to study the relationship between soil chemical properties and abundance of dominant genera. The first two RDA components (RDA1 and RDA2) separated the organic fertilizer treated soils from the chemical fertilizer treated soils (Fig 9). The chemical fertilizer treated samples (NorS) were positively related to the cadmium (Cd), Cuprum (Cu) and plumbum (Pb).

The organic fertilizer treated samples (OrgS and CKOrgS) were positively related to a higher relative abundance of *Catenulispora*, *Candidatus_Solibacter*, *Burkholderia-Paraburkholderia*, *Gemmatirosa*, *Nitrospira*, *Rhizomicrobium* and negatively related to *Acidobacterium*, *Acidothermus* and *Acidicaldus*. Strong associations were found among total nitrogen (TN), total phosphorus (TP), available phosphorus (AP), available nitrogen (AN), cadmium (Cd), Cuprum (Cu) and plumbum (Pb) with the abundance of *Acidothermus*, *Acidobacterium* and *Acidicaldus*. The abundance of *Acidibacter*, *Catenulispora*, *Burkholderia-Paraburkholderia*, *Gemmatirosa*, *Nitrospira*, *Candidatus*_*Solibacter*, *Rhizomicrobium* and *Sorangium* were found to be highly associated with soil pH.

## Discussion

For centuries, the use of organic fertilizer is a common practice to maintain soil fertility and crop yield in China [26–27]. With increased availability of chemical fertilizers since the late 1970s and rise of labor costs since the 1980s, the utilization of organic fertilizer has dramatically declined [26, 28]. In order to prevent food shortage worldwide and maximize crop yield, large amounts of chemical fertilizers have been applied to arable fields over the past few decades [29–30]. However, excessive use of chemical fertilizers has led to several issues such as serious soil degradation, nitrogen leaching, soil compaction, reduction in soil organic matter, and loss of soil carbon. In addition, the efficacy of chemical fertilizers on crop yield has been decreasing over time [29, 31–32]. Because of these concerns, there is a growing demand for development of organic agriculture. In this study, we found that N, P and K contents were not significantly different between organic and chemical fertilizer treatments. These results indicated that organic fertilizers provide similar nutrient elements as chemical fertilizer. In recent years, soil acidification has become a serious problem for modern agriculture in China [33]. The major cause of rising soil acidity is increased use of acidifying nitrogen fertilizers or incomplete cycling of nitrogen species in the soil [33–34]. Our previous studies have found tea orchards soil pH, tea amino acids, and phenolic compounds significantly decreased with increasing years of monoculturing [1, 5]. Furthermore, our data showed that organic fertilizer treatment improved soil pH, and increased the contents of tea polyphenols and amino acids. It is well known that acidification can increase the mobility of heavy metals into the soil where they could be taken up by plants [35–36]. The RDA analysis showed the some acidic microbes were highly associated with heavy metals. Previous studies have verified the heavy metal polluted soil would affect the microbial community structure, microbial biomass and microbial residues [37–39]. Some specific microorganism have the ability to adsorb heavy metal [40–41]. We found that the contents of heavy metals (Cu, Pb, Cd, As) were lower in soil and plant samples under organic fertilizer treatment compared to those treated with chemical fertilizer. The future research should focus the potential mechanism of intrinsic linkages between microbial community and heavy metal under the organic fertilizer treatments.

Healthy and asymptomatic plants in nature are colonized by a rich diversity of microbes, with the complex plant-associated microbial community referred to as a second genome of the plant because of its effect on plant growth and productivity [42–43]. In this study, we found that the long-term use of organic fertilizer significantly increased the bacterial diversity in terms of species richness. Our data are in agreement with the findings of Sun et al. [29], who reported that the NPK chemical fertilizers caused a significant decrease in bacterial diversity. Significant differences in soil bacterial composition were also observed in tea orchards under long-term treatment with chemical or organic fertilizers. The most abundantly identified bacteria phyla were assigned to the *Actinobacteria*, *Chloroflexi*, *Proteobacteria*, and *Acidobacteria* phyla. Similar results were observed with long-term continuous cropping tea orchard systems [5, 44]. Further analysis showed that *Acidothermus*, *Acidobacterium* and *Acidicaldus*, increased significantly in soil samples treated with chemical fertilizer, which are acidophilic and capable of proliferating in an acidic environment. In our RDA analysis, *Acidibacter* were found to be highly associated with soil pH. It has been well documented that soil pH has a marked influence on the composition of the microbial community [45]. Our previous study have shown the plant–microbe interactions contribute to the increased acidity and create a new environment to mediate changes in the microbial community structure in the *R*. *pseudostellariae* rhizosphere under continuous monoculture regimes [46]. We speculate that long-term application of chemical fertilizers decreased soil pH, promoted the proliferation of some specific microbes and activated the heavy metal ions in soil, further deteriorated the physicochemical properties and quality of tea. However, the long-term application of organic fertilizer was able to alleviate some of this negative effect.

Positive plant-soil feedback depends on beneficial interactions between plant roots and microorganisms for growth promotion, nutrient acquisition and disease suppression [4]. Previous studies reported that increasing years of consecutive monoculturing resulted in a significant increase in abundance of pathogens and a decrease in beneficial microorganisms in the rhizosphere of plants [47–48]. The significant decrease in beneficial plant bacteria was also observed in the rhizosphere soil of continuously monocultured tea [5]. In this study, the relative abundance of *Burkholderia*, *Nitrospira*, and *Streptomycetales* was significantly higher with organic fertilizer treatment. Previous studies have found that *Burkholderia* was able to inhibit the growth of pathogens and in effect acted as a Bio-organic fertilizer to improve plant growth [49–50]. *Nitrospira* is a ubiquitous bacterium that has a role in the nitrogen cycle by performing nitrite oxidation in the second step of nitrification [51–52]. Furthermore, *Streptomycetales* can act as PGPR (rhizosphere growth-promoting bacteria) to reduce plant disease and are associated with plant disease suppression in many soils [53–54]. These results suggested that organic fertilizer could improve the soil environment to create a new condition for the growth of potentially beneficial microbes.

In conclusion, long-term application of organic fertilizer treatment will improve the rhizosphere environment in tea orchards. And the organic fertilizer improved tea quality and decreased the level of heavy metals in rhizosphere soil. Furthermore, soil pH and shift in microbiomes were related to fertilizers treatments. Our findings suggest that organic fertilizer can shape microbial composition and recruit beneficial bacteria into the rhizosphere of tea. These results provide a promising strategy to tea orchards by treatment with organic fertilizers.

## Supporting information

S1 Table Relative abundance of the bacterial order among the different samples. (DOCX)

S1 Fig The relative abundance of the bacterial phylum in the four different soil samples. (DOCX)
