# Supplementary material for: The effects of chemical and organic fertilizer usage on rhizosphere soil in tea orchards
Source: PLoS One. 2019 May 28;14(5):e0217018. doi: 10.1371/journal.pone.0217018 (PMC6538140; doi:10.1371/journal.pone.0217018)
Supplement: S1 Table — (DOCX) [file pone.0217018.s001.docx]

The effects of chemical and organic fertilizer usage on rhizosphere soil in tea orchards

Weiwei Lin^1,3^, Manhong Lin^2,3^, Hongyan Zhou^4^, Hongmiao Wu^1,3^, Zhaowei Li^1,3*^, Wenxiong Lin^1,2,3*^

**S1 Table.** Relative abundance of the bacterial order among the different samples

| Order | Relative abundance (%) | | | |
| --- | --- | --- | --- | --- |
|  | NorS | OrgS | CKOrgS | CKNorS |
| *Frankiales* | 22.53a | 12.97c | 17.24b | 21.77a |
| *Rhizobiales* | 5.77a | 4.72b | 4.98ab | 3.20c |
| *Acidobacteriales* | 3.77b | 4.73a | 3.86b | 2.21c |
| *Burkholderiales* | 0.69b | 2.25a | 0.86b | 0.10c |
| *Streptomycetales* | 0.71ab | 1.24a | 0.67ab | 0.56b |
| *Pseudonocardiales* | 0.93ab | 0.49bc | 0.36c | 1.19a |
| *Nitrospirales* | 0.94b | 1.47a | 0.47c | 0.07d |
