# Supplementary material for: The effects of chemical and organic fertilizer usage on rhizosphere soil in tea orchards
Source: PLoS One. 2019 May 28;14(5):e0217018. doi: 10.1371/journal.pone.0217018 (PMC6538140; doi:10.1371/journal.pone.0217018)
Supplement: S1 Fig — (DOCX) [file pone.0217018.s002.docx]

The effects of chemical and organic fertilizer usage on rhizosphere soil in tea orchards

Weiwei Lin^1,3^, Manhong Lin^2,3^, Hongyan Zhou^4^, Hongmiao Wu^1,3^, Zhaowei Li^1,3*^, Wenxiong Lin^1,2,3*^


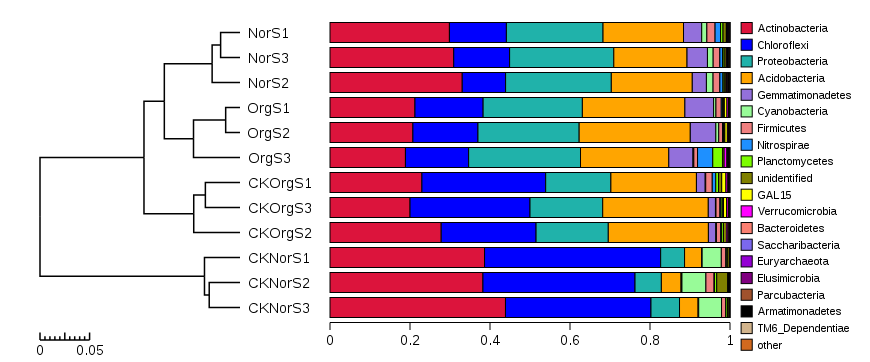


**S1 Fig.** The relative abundance of the bacterial phylum in the four different soil samples.
